# Structural Analysis of the Complex between Penta-EF-Hand ALG-2 Protein and Sec31A Peptide Reveals a Novel Target Recognition Mechanism of ALG-2

**DOI:** 10.3390/ijms16023677

**Published:** 2015-02-06

**Authors:** Takeshi Takahashi, Kyosuke Kojima, Wei Zhang, Kanae Sasaki, Masaru Ito, Hironori Suzuki, Masato Kawasaki, Soichi Wakatsuki, Terunao Takahara, Hideki Shibata, Masatoshi Maki

**Affiliations:** 1 Department of Applied Molecular Biosciences, Graduate School of Bioagricultural Sciences, Nagoya University, Furo-cho, Chikusa-ku, Nagoya 464-8601, Japan; E-Mails: takahashi.takeshi@a.mbox.nagoya-u.ac.jp (T.T.); kojima.kiyousuke@c.mbox.nagoya-u.ac.jp (K.K.); zhang.wei@i.mbox.nagoya-u.ac.jp (W.Z.); guinaijiang@hotmail.co.jp (K.S.); kurukurumegane3310@gmail.com (M.I.); takahara@agr.nagoya-u.ac.jp (T.T.); shibabou@agr.nagoya-u.ac.jp (H.S.); soichi.wakatsuki@stanford.edu (S.W.); 2 Structural Biology Research Center, Photon Factory, Institute of Materials Structure Science, High Energy Accelerator Research Organization (KEK), Tsukuba, Ibaraki 305-0801, Japan; E-Mails: hironori.suzuki@canterbury.ac.nz (H.S.); masato.kawasaki@kek.jp (M.K.); 3 Biomolecular Interaction Centre, School of Biological Sciences, University of Canterbury, Private Bag 4800, Christchurch 8020, New Zealand; 4 Department of Structural Biology, School of Medicine, Stanford University, Stanford, CA 94305-5126, USA

**Keywords:** adaptor protein, calcium-binding protein, COPII, crystal structure, EF-hand, motif, protein-protein interaction

## Abstract

ALG-2, a 22-kDa penta-EF-hand protein, is involved in cell death, signal transduction, membrane trafficking, *etc.*, by interacting with various proteins in mammalian cells in a Ca^2+^-dependent manner. Most known ALG-2-interacting proteins contain proline-rich regions in which either PPYP*X*nYP (type 1 motif) or P*X*PGF (type 2 motif) is commonly found. Previous X-ray crystal structural analysis of the complex between ALG-2 and an ALIX peptide revealed that the peptide binds to the two hydrophobic pockets. In the present study, we resolved the crystal structure of the complex between ALG-2 and a peptide of Sec31A (outer shell component of coat complex II, COPII; containing the type 2 motif) and found that the peptide binds to the third hydrophobic pocket (Pocket 3). While amino acid substitution of Phe^85^, a Pocket 3 residue, with Ala abrogated the interaction with Sec31A, it did not affect the interaction with ALIX. On the other hand, amino acid substitution of Tyr^180^, a Pocket 1 residue, with Ala caused loss of binding to ALIX, but maintained binding to Sec31A. We conclude that ALG-2 recognizes two types of motifs at different hydrophobic surfaces. Furthermore, based on the results of serial mutational analysis of the ALG-2-binding sites in Sec31A, the type 2 motif was newly defined.

## 1. Introduction

ALG-2 (apoptosis-linked gene 2, gene name: *PDCD6*), a 22-kDa Ca^2+^-binding protein, contains five serially-repeated EF-hand motifs (EF1 to EF5) and belongs to the penta-EF-hand (PEF) protein family, including typical calpains, sorcin, grancalcin and peflin (see [1] for a review). Since ALG-2 is evolutionarily conserved from lower eukaryotes to mammals, in contrast to restricted conservation in higher eukaryotes in the cases of other PEF proteins, ALG-2 is regarded as a prototype of PEF proteins [1,2]. While the PEF domains of typical calpains regulate proteolytic activity, other PEF proteins lack catalytic activities and are thought to exert their biological functions by interacting with intracellular proteins. Although the precise mechanisms have remained unclear, ALG-2 has been suggested to be involved in diverse cellular functions, such as apoptosis [3,4,5,6], cancer development [7,8], signal transduction [9,10], membrane trafficking [11,12,13,14] and post-transcriptional control [15,16]. Upon binding to Ca^2+^, ALG-2 changes its conformation and interacts with various intracellular proteins containing Pro-rich regions, including ALIX and HD-PTP (auxiliary proteins of the endosomal sorting complex required for transport, ESCRT), TSG101 and VPS37s (ESCRT-I subunits), annexins A7 and A11 (AnxA7 and AnxA11; Ca^2+^-dependent phospholipid-binding proteins), Sec31A (see below), PLSCR3 (a Tubby-like protein superfamily member), scotin (a p53-induced ER transmembrane protein), PATL1 (a component of RNA processing body, P-body) and RBM22 and CHERP (splicing modulation factors); see [17,18] and the references therein.

Sec31, conserved from yeast to humans, is a component of the outer layer coat protein complex II (COPII) and is enriched in the endoplasmic reticulum (ER) exit sites, from which cargoes contained in COPII vesicles are transported from the ER to the Golgi apparatus [19,20]. ALG-2 binds one of the two mammalian isoforms encoded by different genes, designated Sec31A, in a Ca^2+^-dependent manner and is recruited to the ER exit sites [11,12,13]. Although the exact molecular mechanism is still not clear, ALG-2 has been shown to be involved in regulation of ER to Golgi transport at least at two steps by *in vitro* assays, including recombinant ALG-2 proteins: suppression of homotypic fusion of COPII vesicles using high-speed cellular supernatants of conditioned cells [14] and attenuation of the budding of COPII vesicles using purified COPII proteins and permeabilized cells [21]. Leaking luminal Ca^2+^ from the ER or vesicles has been suggested to be the source of Ca^2+^ utilized by ALG-2 in the ER to Golgi transport regulation associated with COPII [14,22]. The observed effects of ALG-2 depletion by siRNA on the transport of cargoes, however, are controversial: no apparent effects (data not shown in [11]), mild suppressive effects (figure not shown in [22]) and accelerating effects [23]. The discrepancies are probably due to differences in methods, assay conditions and cell lines used.

ALIX was first identified as an ALG-2-interacting protein [24,25]. We previously identified ALG-2-binding sites in the Pro-rich regions of ALIX [26,27], PLSCR3 [28] and Sec31A [29]. Comparison of these binding sequences led us to define two ALG-2-binding motifs: type 1, PPYP*X*nYP (*X*, variable; *n* = 4 in ALIX and PLSCR3); type 2, P*X*PGF (Sec31A and PLSCR3; F substitutable with W in PLSCR3) [17,28]. Notably, PLSCR3 has both type 1 and type 2 motifs. While ALG-2^ΔGF122^ (an alternatively spliced shorter isoform lacking Gly^121^Phe^122^) does not bind ALIX, it binds Sec31A and PLSCR3, suggesting the presence of at least two different modes of interactions between ALG-2 and its binding partners [28]. X-ray crystal structural analysis of the complex between ALG-2 and ALIX peptide has revealed that a 16-residue ALIX peptide binds ALG-2 at two juxtaposed hydrophobic pockets, designated Pocket 1 and Pocket 2, that accommodate PPYP and the *C*-terminal YP, respectively [27]. To address the question of whether the type 1 and type 2 motifs share the binding pockets in the ALG-2 molecule, we previously searched for a hydrophobic cavity in ALG-2 by a computational algorithm and predicted a new ligand-binding site [30]. The predicted hydrophobic pocket, corresponding to Pocket 3, located away from Pocket 1 and Pocket 2, fit with *N*-acetyl-^1^ProAlaProGlyPhe^5^-amide, a virtual penta-peptide derived from the type 2 motif ALG-2-binding site in PLSCR3, in a docking simulation program. However, a line of solid experimental evidence has been needed to corroborate the Pocket 3-type 2 motif hypothesis. In the present study, we resolved the crystal structure of the complex between ALG-2 and a 12-residue Sec31A peptide and found that the type 2 motif peptide indeed binds Pocket 3. Different effects of site-specific mutagenesis of ALG-2 on binding to endogenous Sec31A and ALIX proteins by glutathione-*S*-transferase (GST)-pulldown assays using cell lysates also suggest the utilization of different hydrophobic surfaces for the interaction with Sec31A and ALIX. Furthermore, we refined the type 2 motif by interaction analysis using various mutants of the Sec31A peptide and other known or potentialALG-2-interacting proteins. The adaptor function of ALG-2 in the COPII system is also discussed.

## 2. Results

### 2.1. Co-Crystallization

**Figure 1:**
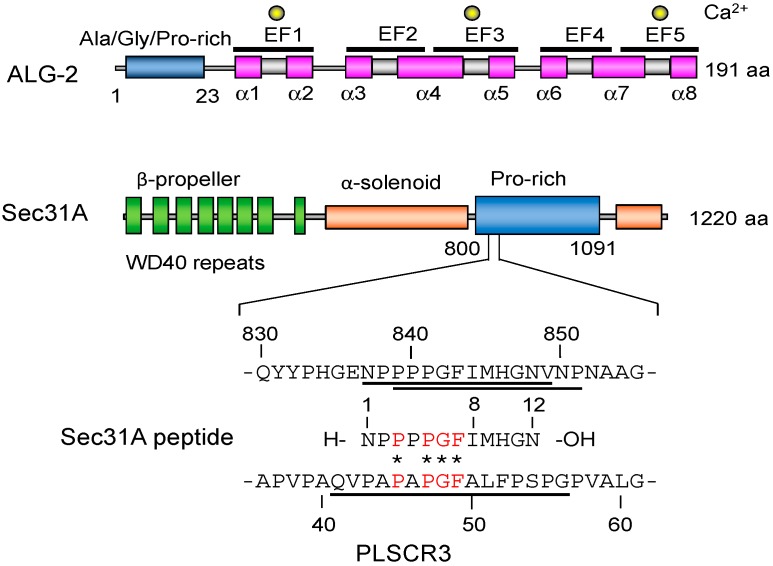
Schematic representation of ALG-2 and Sec31A. ALG-2 has an *N*-terminal Ala/Gly/Pro-rich flexible region and a penta-EF-hand (PEF) domain containing a unique feature of five EF hands (EF1–5) with eight α-helices (α1–8). An *N*-terminally truncated mutant (des3-20ALG-2) was bacterially expressed and used for crystallization. Calcium ions (Ca^2+^) bind to EF1, EF3 and EF5 by EF-hand Ca^2+^-coordination. Sec31A contains WD40 repeats and a Pro-rich region. Previously identified ALG-2-binding sites indicated by underlines in Sec31A and PLSCR3 have a conserved P*X*PGF motif indicated by asterisks and letters in red color [28,29]. A synthetic 12-residue Sec31A peptide was used for co-crystallization with the recombinant des3-20ALG-2 protein.

ALG-2 has a flexible *N*-terminal Ala/Gly/Pro-rich region. In our previous study, we purified bacterially expressed recombinant *N*-terminal truncated ALG-2 (des3-20ALG-2 and des3-23ALG-2) protein that had been successfully used for the crystallization of apo-form and co-crystallization with the ALIX peptide, respectively [27]. These two types of *N*-terminal truncated ALG-2 were used for co-crystallization with the human Sec31A peptide (837–848 or 835–850), which contains a type 2 motif (P*X*PGF) and is partially conserved with the ALG-2-binding site in PLSCR3 (Figure 1).

The co-crystals of des3-20ALG-2/Sec31A 837–848 obtained in the presence of Zn^2+^ belonged to space group *P*6_4_, and the crystals were twinned. The crystal structure was resolved at 2.4 Å resolution. Data collection, processing and refinement statistics are summarized in Table S1 (PDB code: 3WXA). Other attempts of co-crystallization in different combinations, including the one in the presence of Ca^2+^, were not successful.

### 2.2. Overall Structure of the Complex between ALG-2 and Sec31A Peptide

**Figure 2:**
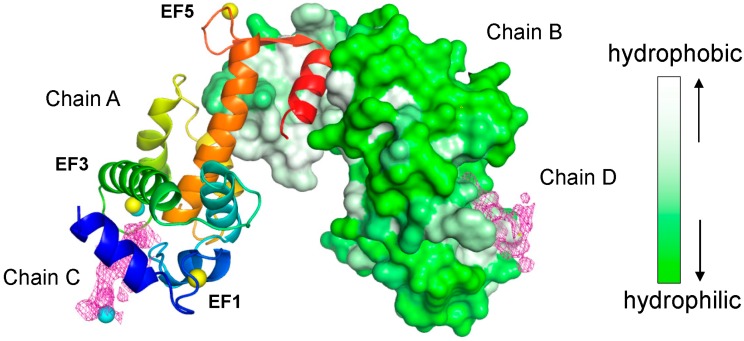
X-ray crystal structure of the complex between ALG-2 and the Sec31A peptide. The overall structure of the complex. Chain A and chain B (ALG-2 molecules) are shown by cartoon (rainbow color: blue in the *N*-terminal region and red in the *C*-terminal region) and surface representation (gray-white, hydrophobic; green, hydrophilic), respectively, using PyMOL software. Chain C and chain D, illustrating the electron density of the peptide, are shown in magenta mesh. Yellow spheres and cyan spheres indicate EF-hand coordinated and non-EF-hand coordinated zinc ions, respectively, in chains A and C.

An asymmetric unit of the crystal contained two ALG-2 molecules (chains A and B) as a dimer and two Sec31A peptides (NPPPPGFIMHGN, chains C and D). Figure 2 shows a cartoon representation of chain A and a surface representation of chain B, as well as an electron density map corresponding to Sec31A peptides (purple mesh). Three Zn atoms (yellow spheres) are found in the Ca^2+^-coordinating sites in EF1, EF3 and EF5, essentially as described previously [27,31,32], and two additional Zn atoms (cyan spheres) are found in the vicinities of EF3 and the Sec31A peptide in each chain.

### 2.3. Structure of the Sec31A Peptide

**Figure 3:**
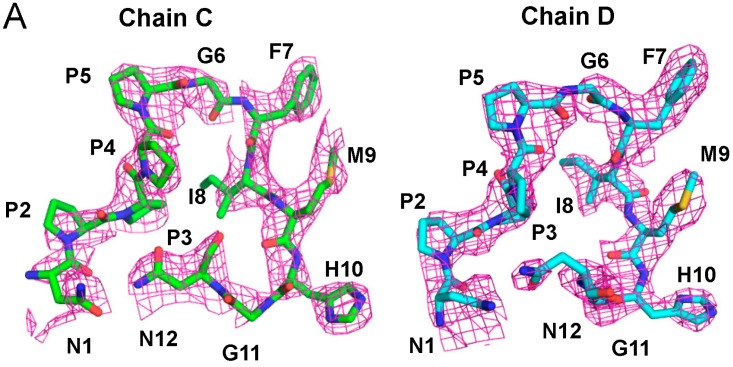

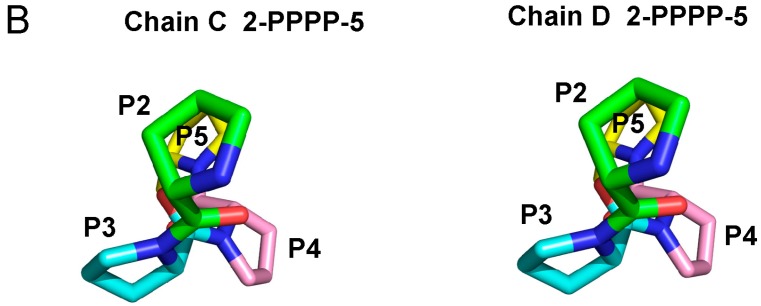
(**A**) Stick models of chains C and D are superposed with an electron density map shown in mesh. In the stick model, carbon atoms are shown in light green in chain C and in cyan in chain D, and nitrogen, oxygen and sulfur atoms are shown in blue, red and yellow, respectively, in both chains; (**B**) Left-handed type II polyproline (PPII) helices are formed in the Pro-clustered segments (^2^PPPP^5^) in chains C and D. Pro residues are highlighted in different colors as indicated (from green, cyan, pink to yellow, in order).

The constructed stick models of chains C and D, superposed with an electron density map in mesh, show structures that are similar to each other from Pro^2^ to His^1^^0^ (Figure 3A). The clustered Pro residues (^2^PPPP^5^) form left-handed type II polyproline (PPII) helices (Figure 3B). A short left-handed PPII helix and surrounding non-proline residues are known to be important for signaling proteins to be specifically recognized by SH3 domains [33,34]. A group of WW domains also recognize PPII helical peptides [35].

### 2.4. Binding of the Sec31A Peptide to Pocket 3

**Figure 4:**
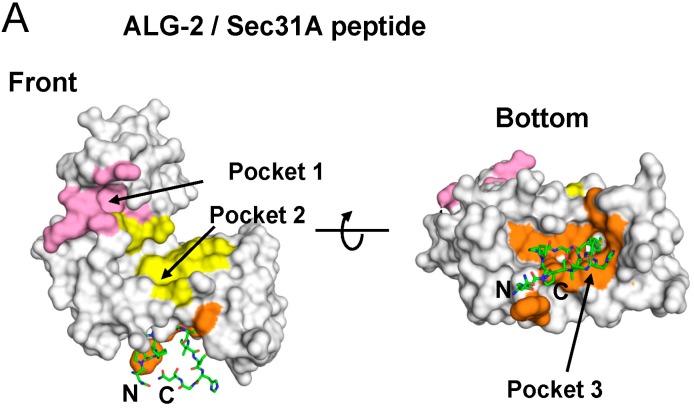

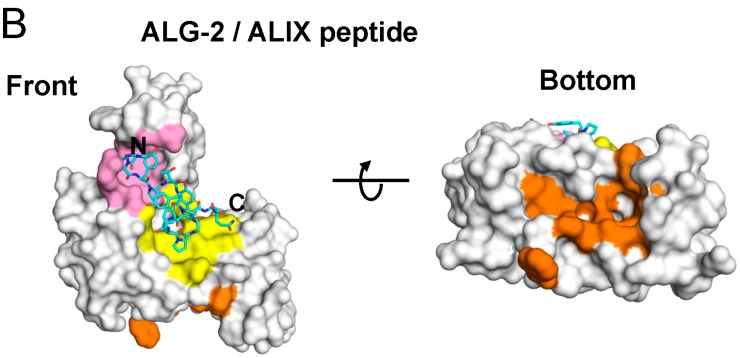
Different hydrophobic pockets used for binding to Sec31A and ALIX. While the Sec31A peptide (**A**, shown in the stick model; carbons colored in green) binds to Pocket 3 (orange), the ALIX peptide (**B**, shown in the stick model; carbons colored in cyan) binds to adjacent Pocket 1 (**B**, pink) and Pocket 2 (**B**, yellow). Unrelated areas are shown by gray surfaces. (**A**) Complex between ALG-2 and the Sec31A peptide (PDB code: 3WXA; chains A and C); (**B**) complex between ALG-2 and the ALIX peptide (PDB code: 2ZNE; chains A and C).

**Figure 5:**
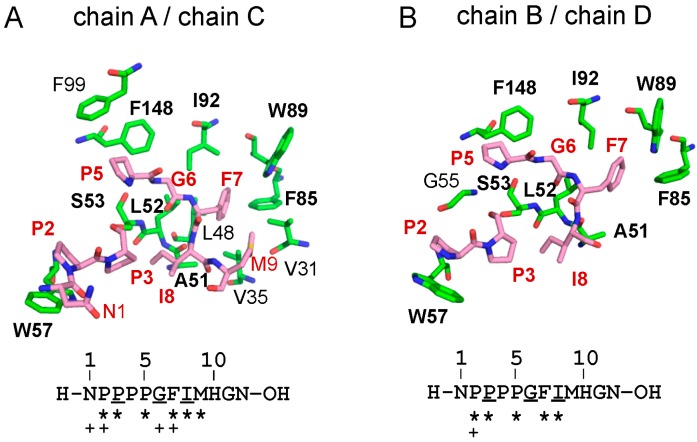
Interactions between ALG-2 Pocket 3 residues and Sec31A peptide. Residues involved in interactions between ALG-2 and the Sec31A peptide (**A**, chains A and C; **B**, chains B and D) are shown in stick models (ALG-2, green; Sec31A peptide, pink). Nitrogen and oxygen atoms are shown in blue and red, respectively. ALG-2 residues commonly interacting with the peptides in both chains A and C are indicated in bold face. Asterisks and plus symbols indicate side chain and main chain carbon atoms of the Sec31A peptide residues that interact with side chain atoms of ALG-2 molecules, respectively. Underlines indicate residues involved in hydrogen bonding.

The peptide ligand binds to a hydrophobic pocket designated Pocket 3, which is separated from the ALIX-binding pockets, named Pocket 1 and Pocket 2 (Figure 4, pink and yellow surfaces) [27]. Pocket 3 (orange) is formed by residues that are present in EF1 (F27, V31, V35, L48, A51), the EF1–EF2-connecting loop (L52, S53, G55, W57), EF2 (F85, V88, W89, I92), EF3 (T93, Q96, F99, G108, M109) and EF4 (F148). As shown in Figure 5 and Figure S1, several Pocket 3-forming residues interact with the Sec31A peptide at the side chain or main chain carbon atoms of the peptide by hydrophobic interactions (common contact residues in both chains B and D are indicated in bold face: A51, L52, S53, W57, F85, W89, I92, F148). Hydrogen bonds are formed between chains A and C (S53^OG^/P3^O^, S53^O^/G6^N^, S53^N^/G6^O^, A51^O^/I8^N^) and between chains B and D (S53^OG^/P3^O^, S53^N^/G^6O^, A51^O^/I8^N^).

#### 2.4.1. Analysis of the Ca^2+^-Dependent Interaction of Sec31A by GST-Pulldown

**Figure 6:**
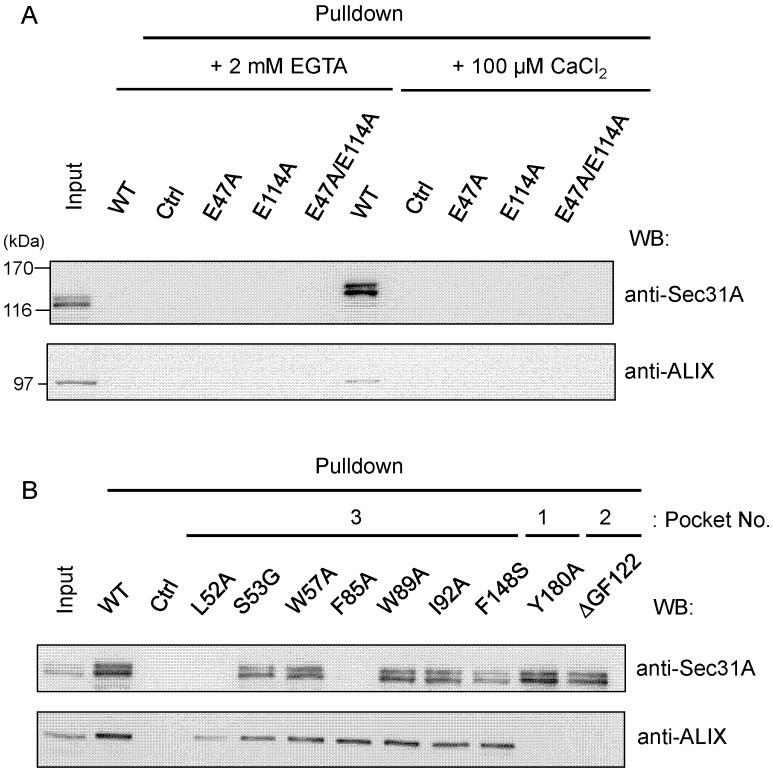
Glutathione-*S*-transferase (GST)-pulldown assays of ALG-2 mutants. Endogenous Sec31A and ALIX in ALG-2-knockdown HEK293 cells (HEK293/ALG-2_KD_ cells) were pulled down with GST-fused wild-type (WT) and mutant ALG-2 proteins of (**A**) EF-hands (*E47A*, EF1; *E114A*, EF3; *E47A*/*E114A*, EF1 and EF3) and (**B**) hydrophobic pockets (Pocket 1, *Y180A*; Pocket 2, *ΔGF122*; Pocket 3, *L52A*, *S53G*, *W57A*, *F85A*, *W89A*, *I92A*, *F148S*). Unfused GST was used as a negative control (Ctrl). Cleared cell lysates were incubated with glutathione Sepharose beads immobilizing GST-fusion proteins in the presence of (**A**) 2 mM EGTA or (**A**,**B**) 100 μM CaCl_2_, as described in the Experimental Section. Proteins bound to the beads (pulldown) were resolved by SDS-PAGE (7.5% and 10% gels for Sec31A and ALIX analyses, respectively) and transferred to PVDF membranes for Western blotting (WB) with antibodies against Sec31A and ALIX, as indicated. The relative amount of cleared cell lysate proteins (input) used for analysis of pulldown products was 12.5%. Representative data obtained from three independent experiments are shown.

To evaluate the individual amino acid residues in ALG-2 for contribution to the interactions with the Sec31A protein, we performed GST-pulldown assays using cleared cell lysates of previously established HEK293/ALG-2_KD_ cells in which expression of endogenous ALG-2 had been suppressed by the RNA interference method [36]. Proteins were pulled down with GST proteins that were fused with wild-type (WT) or with various mutant ALG-2 proteins. The pulldown products were resolved by SDS-PAGE, followed by Western blotting (WB) using antibodies against Sec31A or ALIX. First, the effects of Ca^2+^ on the interactions by the GST-pulldown assay were investigated. As shown in Figure 6A, WB signals were detected for both Sec31A and ALIX in the pulldown products of GST fused with wild-type (WT) ALG-2 in the presence of 100 μM CaCl_2_, but not in the presence of 2 mM EGTA. WB signals were not detected in the pulldown products of unfused control GST (Ctrl), even in the presence of 100 μM CaCl_2_, indicating a specific Ca^2+^-dependent interaction between ALG-2 and its binding partners (Sec31A and ALIX). The Ca^2+^-dependency of the pulldown efficiency was also investigated by using mutants of Ca^2+^-coordinating positions of EF1 (E47A), EF3 (E114A) and both EF1 and EF3 (E47A/E114A). All of these EF hand mutants lost binding abilities.

#### 2.4.2. Identification of Critical Pocket 3 Residues for Interaction

Next, the contributions of interacting residues in ALG-2 were investigated by mutagenesis. Among amino acid substitution mutants of Pocket 3 in ALG-2, WB signals of Sec31A were reduced significantly in the mutants of L52A and F85A and slightly in F148S, but no significant adverse effects were observed in the mutants of W57A, W89A and I92A under the conditions used in the presence of 100 μM CaCl_2_ (Figure 6B). The S53G mutant had only a small effect on binding to Sec31A, suggesting that the contribution of hydrogen bonding by the gamma oxygen atom of Ser^53^ is small for the interaction with Sec31A. All Pocket 3 mutants examined in the present study retained binding abilities to ALIX, except for the mutant of L52A, which showed a small reduction. The mutants of *Y180A* (Pocket 1) and *ΔGF122* (Pocket 2, deletion of residues Gly^121^Phe^122^) showed opposite effects, *i.e.*, loss of binding to ALIX, but little effects on Sec31A binding.

### 2.5. Co-Immunoprecipitation Assays of Sec31A Mutants

**Figure 7:**
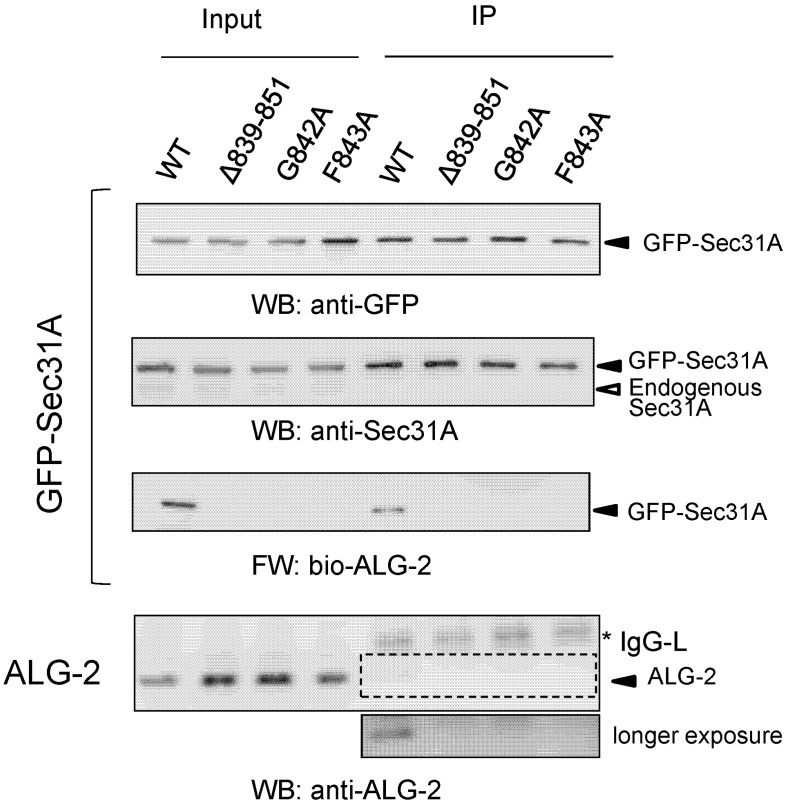
Co-immunoprecipitation assays of green fluorescent protein (GFP)-fused Sec31A mutants. At 24 h after HEK293T cells had been transfected with expression plasmids for GFP-Sec31A encoding wild-type (WT), ALG-2-binding site deletion mutant (*Δ839*–*851*) or amino acid substitution mutants (*G842A*, *F843A*), cells were lysed, and the obtained cleared cell lysates were subjected to immunoprecipitation with rabbit antiserum against GFP, as described in the Experimental Section. Cleared cell lysates (input) and immunoprecipitates (IP) were subjected to WB with anti-GFP (top) and anti-Sec31A (second row) and far-Western blot (FW) with biotin-labeled ALG-2 (bio-ALG-2, third row), as indicated. WB was also performed with anti-ALG-2 (bottom), and the area surrounded by the broken line indicates an image of longer exposure time for chemiluminescence reaction. Asterisk, immunoglobulin light chain (IgG-L). Input, 2.5%. Representative data obtained from three independent experiments are shown.

Since Phe^85^ of ALG-2 interacts hydrophobically with Phe^7^ in the 12-residue Sec31A peptide in the crystal structure, we investigated the importance of Phe^843^ (corresponding to Phe^7^ in the peptide) and its neighboring residue Gly^842^ in the full-length Sec31A protein. Green fluorescent protein (GFP)-fused Sec31A proteins of amino acid-substituted mutants (G842A and F843A), as well as a positive control (WT) and a negative control (deletion mutant of the entire ALG-2-binding site, *Δ839*–*851*) were expressed in HEK293T cells and immunoprecipitated with antiserum against GFP, and the immunoprecipitates (IP) were analyzed by WB and far-Western (FW) (Figure 7). Despite equal amounts of loaded GFP-Sec31A proteins, as indicated by similar signal intensities by anti-GFP and anti-Sec31A, all three mutants had no signals in FW with biotin-labeled ALG-2 (bio-ALG-2) and WB with anti-ALG-2.

### 2.6. Identification of Critical Residues in Sec31A for Binding to ALG-2

**Figure 8:**
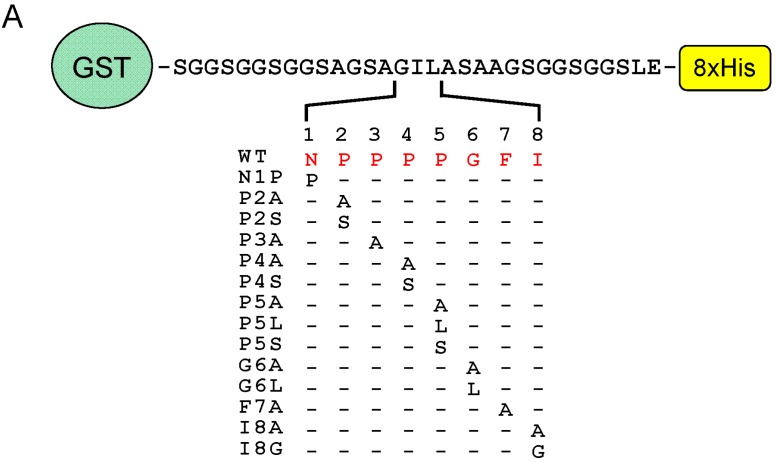

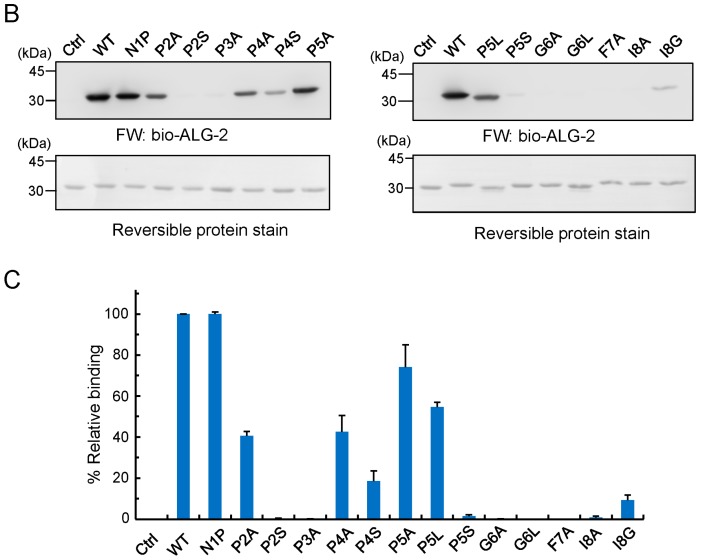
Determination of critical residues in the Sec31A peptide sequence for ALG-2 interactions. (**A**) Amino acid sequences of the GST-fusion vector in the cloning site and mutant Sec31A peptides. *Escherichia coli* expression plasmids for amino acid-substituted mutants of eight-residue Sec31A peptides were generated by inserting each synthetic oligonucleotide block into a GST-SGG-linker-8xHis vector; (**B**) Far-Western blot analysis. Affinity-purified control GST (Ctrl) and Sec31A peptide-fused GST proteins of wild-type (WT) and mutants were resolved by SDS-PAGE, transferred to PVDF membranes and stained with a reversible protein stain kit (**lower** panels). After being destained, the membranes were subjected to FW by probing with bio-ALG-2 (**upper** panels). Representative data from four independent experiments are shown; (**C**) Relative binding activities of mutants. The signal intensity of FW was measured with image processing software ImageJ. Relative binding activities compared to WT were calculated and expressed in percentages (mean ± S.E.; *n* = 4).

To further investigate which residues in the Sec31A peptide are important for binding to ALG-2, we performed far-Western blot (FW) of recombinant GST-SGG-linker-8xHis proteins that were fused with an eight-residue Sec31A peptide (NPPPPGFI) and its amino acid-substituted mutants (Figure 8A) by using bio-ALG-2 as a probe. Regardless of the equal amounts of loaded proteins between WT and mutants, as indicated by reversible protein stain before subjecting to FW (Figure 8B, lower panels), FW signals were barely detectable for the mutants of P2S, P3A, P5S, G6A, G6L, F7A and I8A (Figure 8B, upper panels). While Pro^3^, Gly^6^, Phe^7^ and Ile^8^ were not substitutable with Ala, substitution of Pro with Ala at position 2, 4 or 5 (P2A, P4A or P5A) or substitution of Pro^5^ with Leu (P5L) retained the binding activities of approximately 40% to 75% of WT, as shown in the quantified data (Figure 8C). A complete loss of the activity by substitution of Pro at positions 2 or 5 with Ser (P2S or P5S) suggests that these positions need to be hydrophobic for acquiring stronger interactions with ALG-2. On the other hand, substitution of Pro^4^ with Ser (P4S) retained about 20% of the activity, in agreement with the fact that Pro^4^ does not interact with ALG-2 (Figure 5), supporting the variability of this position.

### 2.7. Comparison of ALG-2-Binding Motif Type 2 Sequences

**Figure 9:**
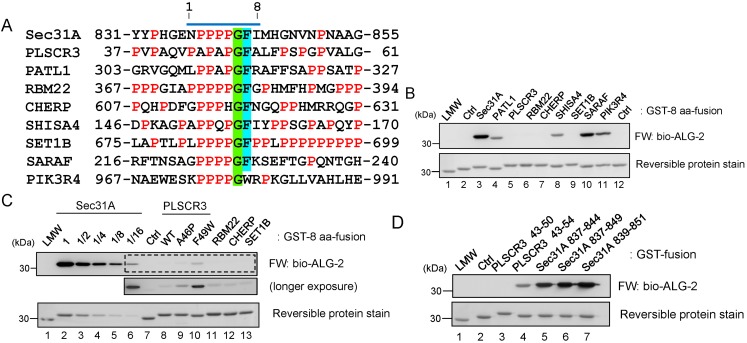
Comparison of ALG-2-binding motif type 2 sequences. (**A**) Amino acid sequences of known and potential ALG-2-binding proteins. The sequence of the ALG-2-binding site in Sec31A is compared with similar sequences from other known ALG-2-binding proteins (PLSCR3, PATL1, RBM22, CHERP) or with those from proteins whose interactions with ALG-2 have not yet been determined (SET1B, SARAF, PIK3R4) or need to be confirmed (SHISA4). Prolines are highlighted in red. Conserved glycines and phenylalanines are highlighted in light-green and cyan, respectively. A bar above the Sec31A sequence indicates an 8-aa segment used for the preparation of GST-fused peptides shown in (**B**) (GST-8 aa-fusion); (**B**) FW analysis. ALG-2-binding activities of GST-8 aa-fusion proteins were determined by FW with bio-ALG-2. Lane 1, low molecular weight (LMW) marker proteins; Lanes 2 and 12, control GST (Ctrl); Lanes 3–11, GST-fused peptides containing 8-aa sequences shown in (**A**). **Top**, FW, exposure time of 10 s; **bottom**, reversible protein stain; (**C**) FW analysis of weak binding proteins. The GST 8-aa fusion protein of Sec31A was serially diluted to decrease the FW signal intensity. An image of longer exposure time (130 s) of chemiluminescence reaction is presented to show positive signals for other proteins in the area surrounded by the broken line in the top panel (longer exposure); (**D**) The ALG-2-binding activities of longer PLSCR3 and Sec31A peptides. ALG-2-binding activities of GST-fusion proteins of longer peptides of PLSCR3 (Lane 3, 8 aa, encoding 43–50; Lane 4, 12 aa, encoding 43–54) and Sec31A (Lane 5, 8 aa, encoding 837–844; Lane 6, 13 aa, encoding 837–849; Lane 7, 13 aa, encoding 839–851) were analyzed by FW with bio-ALG-2. Lane 1, low molecular weight (LMW) marker proteins; Lane 2, negative control.

To gain more insight into the binding motif, we compared the binding activities of GST-fusion peptides containing sequences derived from other known ALG-2-binding proteins (PLSCR3, PATL1, RBM22 and CHERP) or P*X*PG[FW]-containing sequences retrieved from databases (SHISA4, SET1B, SARAF and PIK3R4) (Figure 9A). By FW analysis, strong signals were observed in Sec31A and SARAF with similar intensities and in PIK3R4, PATL1 and SHISA4 with slightly reduced intensity (Figure 9B). The signal for PLSCR3, RBM22, CHERP and SET1B was weaker than 1/16 of that for Sec31A by serial dilution (Figure 9C). Substitution of PLSCR3 residues at Ala^46^ with Pro significantly increased the signal intensity, and substitution of Phe^49^ with Trp also had an enhancing effect (Figure 9C). Extending the PLSCR3 peptide (from the sequence encoding 43–50, 8 aa, to 43–54, 12 aa) also significantly enhanced the signal, whereas this was not evident for the Sec31A peptide, which already exhibited a stronger activity (from the sequence encoding 837–844, 8 aa, to 837–849 or 839–851, 13 aa) (Figure 9D). Residues neighboring the motif may also contribute to the enhancement of the interaction.

## 3. Discussion

ALG-2 binds Ca^2+^-dependently to a variety of cellular proteins, most of which contain Pro-rich regions, and binding motifs are classified into type 1 (PPYP*X*nYP) and type 2 (P*X*PGF) [17,18,28]. X-ray crystal structural analysis of ALG-2 was first done with a Ca^2+^-bound form of the recombinant mouse ALG-2 protein by the joint group of Cygler and Berchtold, and it was shown that ALG-2 also forms a dimer by pairing the *C*-terminal EF5 region like other PEF proteins, such as classical calpains and grancalcin [31]. Seven years later, we resolved the X-ray crystal structures of the recombinant human proteins in different forms: a metal-free form, a Ca^2+^-bound form, a Zn^2+^-bound form and a Zn^2+^- and ALIX peptide-bound form [27,37]. The ALIX peptide, containing a type 1 ALG-2-binding motif (PPYP*X*nYP), binds ALG-2 at Pocket 1 and Pocket 2 (Figure 4B). In this study, we resolved the structure of ALG-2 bound with a 12-residue Sec31A peptide containing a type 2 ALG-2-binding motif (P*X*PGF). The Sec31A peptide binds ALG-2 at a hydrophobic pocket, designated Pocket 3, formed by residues that are present from EF1 to EF3, except for F148 (the loop in EF4) (Figure 4A and Figure 5). This pocket is completely separated from Pockets 1 and 2, which are formed by residues from EF2 to EF5 (Figure 4B).

Although the crystals of the ALG-2/Sec31A peptide complex were successfully obtained only in the presence of Zn^2+^ and not in the presence of Ca^2+^, the resolved structure of the Zn^2+^-bound form may mimic the structure of the Ca^2+^-bound form. In the crystal structures, zinc ions are coordinated at EF-hands in a manner similar to the calcium ions in ALG-2 (Figure 2 and [27,32]). We previously showed that Zn^2+^, not Mg^2+^, was also effective for ALG-2 to bind its target protein [27]. However, the required concentration of Zn^2+^ was as high as 100 μM in contrast to an effective concentration of Ca^2+^ as low as 5 μM [28]. Although Zn^2+^-dependent interactions of ALG-2 with target proteins seem non-physiological, we cannot exclude the possibility of the formation of a complex between ALG-2/Zn^2+^ with Sec31A or with other proteins under pathological conditions.

The effects of mutations in ALG-2 on binding to Sec31A and ALIX by GST-ALG-2 pulldown assays in the presence of Ca^2+^ were different between the two proteins (loss of binding to Sec31A: L52A and F85A; loss of binding to ALIX: Y180A and ΔGF122) (Figure 6B). The side chains of Leu^52^ and Phe^85^ in Pocket 3 of ALG-2 interact with those of Pro^5^ and Phe^7^, respectively, in the Sec31A peptide (corresponding to Pro^841^ and Phe^843^, respectively, in the Sec31A protein) (Figure 5). Substitution of Leu^52^ with Ala also exhibited a small adverse effect on binding to ALIX (Figure 6B). This may be explained by the fact that Leu^52^ interacts intra-molecularly with Phe^60^, which is a highly conserved residue in all PEF proteins [38], and F60A lost the ability to bind both Sec31A and ALIX [28]. Thus, Leu^52^ may contribute to the stability of the PEF protein structure. We previously proposed a Ca^2+^/EF3-driven arginine switch mechanism by which binding of Ca^2+^ to EF3 enables the side chain of Arg^125^, present in the loop connecting EF3 and EF4, to move enough to make Pocket 1 accessible to the critical PPYP motif of ALIX [27]. On the other hand, no conspicuous differences were found in the structure of Pocket 3 between the structures of the metal-free and Ca^2+^ or Zn^2+^-bound forms (Figure S2), and the structural basis of the Ca^2+^-dependency of ALG-2 for Sec31A binding could not be elucidated in the present study. Since Pocket 3 is formed by residues in EF1 to EF3, the loss of binding abilities to Sec31A in the mutants of the Ca^2+^-binding loops of EF1 (E47A) and EF3 (E114A) (Figure 6A) may be due to structural changes of Pocket 3 itself in addition to the inability to bind Ca^2+^.

By comparing the ALG-2-binding sites in Sec31A and PLSCR3, as well as the substitutability of Phe with Trp in the binding assay [28], we previously proposed P*X*PG[FW] (*X*, variable) as ALG-2-binding motif type 2 [18,28]. In this study, we newly propose the seven-residue type 2 motif for optimum binding: [PΦ]P*X*[PΦ]G[FW]Ω ([PΦ], Pro or hydrophobic; [FW], Phe or Trp; Ω, large side chain; *X*, variable). Substitution of the conserved Gly with Ala in GFP-Sec31A (Figure 7, G842A) and in the GST-fusion peptide (Figure 8B, G6A) abolished the binding activity. Since Gly has a hydrogen atom as its side chain instead of a β carbon atom and since there is much more conformational flexibility than other amino acid residues in the protein structure, the Sec31A peptide may well fit into Pocket 3 without steric hindrance. Refinement of the type 2 motif should enable the prediction of novel ALG-2-binding proteins with increased reliability.

**Figure 10:**
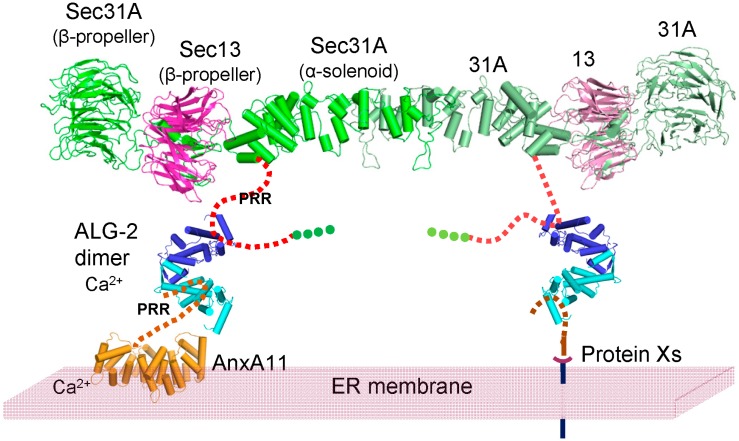
Model for the adaptor function of ALG-2 in anchoring the outer shell of coat complex II (COPII) at the endoplasmic reticulum (ER) membrane. An ALG-2 dimer (cyan and dark blue) bridges the assembly unit of the outer COPII shell and annexin A11 (AnxA11) on the ER membrane by binding a Pro-rich region (PRR) of Sec31A (red broken line) and that of AnxA11 (orange broken line) in the presence of Ca^2+^. An ALG-2 dimer may also directly or indirectly bind unknown proteins (Protein Xs, brown broken line representing the PRR of a putative ALG-2-interacting protein and black line representing an ER-transmembrane tethering protein) on the ER membrane to facilitate anchoring of the COPII coat. For simplification of the diagram, the Sec23/Sec24 inner coat cage is not shown, and only one heterotetrameric unit of Sec31A (green and pale green) and Sec13 (magenta and pink) is illustrated. A 3D-structural model of human Sec31A (UniProt: O94979) was generated by the SWISS-MODEL program using a yeast Sec31/Sec13 heterotetramer model ([43]; PDB code: 4BZJ, chain A) as a template for the *N*-terminal β-propeller and central α-solenoid domains. The Pro-rich region (PRR, broken red line) and the *C*-terminal α-solenoid domain (green dotted line) are predicted to be outside the architectural core of the assembly unit [44]. The β-propeller structure of human Sec13 is taken from PDB Code 3BG0, chain A. The core domain of human AnxA11 (UniProt: P50995) was modeled using the bovine AnxA4 structure (PDB code: 1ANN) as a template.

Since each molecule of the ALG-2 dimer has binding pockets for both type 1 and type 2 motifs, ALG-2 functions as a Ca^2+^-dependent adaptor to bridge ALIX and ESCRT-I components, including TSG101 and VPS37 isoforms, in which type 1 motifs are used for binding [36,39,40]. Annexin A11 (AnxA11) has a sequence similar to the type 1 motif (^4^PGYPPPPGGYPP^15^), and the binding profile of ALG-2 mutants shows a similarity to binding to ALIX [28]. We have recently shown that ALG-2 bridges Sec31A and AnxA11 to attenuate the transport of cargoes from the ER to Golgi [23]. In the experiments performed in parallel, however, ALG-2 did not bridge Sec31A and ALIX and showed a specificity in the adaptor function. Subcellular localization of each binding protein seems important to be bridged by ALG-2. The outer coat cage of COPII has been reconstituted with Sec13 and Sec31 *in vitro* and is self-assembled into a cuboctahedron with 24 units of Sec13/31 heterotetramers, as modeled by cryo-electron microscopy [41,42,43] and X-ray crystallography [44]. Figure 10 shows a model for the adaptor function of ALG-2 in anchoring the outer COPII coat at the ER membrane. An ALG-2 dimer bridges the assembly unit of the outer COPII coat and AnxA11 on the ER membrane by binding a Pro-rich region (PRR) of Sec31A and that of AnxA11 in the presence of Ca^2+^. An ALG-2 dimer may also directly or indirectly bind unknown proteins containing either the type 1 or type 2 motif on the ER membrane to facilitate the anchoring of the COPII coat.

The Ca^2+^-dependent binding of ALG-2 to Sec31A or to its orthologous proteins seems restricted to higher vertebrates. Evolutional comparison of the amino acid sequences of Sec31 proteins reveals that conservation of the type 2 motif is restricted to higher vertebrates, including reptiles, birds and mammals (Figure S3). The budding yeast, *Saccharomyces cerevisiae*, has a PEF protein named Pef1p [45]. Interestingly, Pef1p has been shown to bind yeast Sec31p at the Pro-rich region in the absence of Ca^2+^, and the complex dissociates by inclusion of Ca^2+^ in the binding assay mixture [46]. The reverse interaction―Ca^2+^-dependent dissociation of a PEF protein from the binding target―has been shown for the interaction of grancalcin with l-plastin, a leukocyte-specific actin-bundling protein [47]. Since annexins are absent in the budding yeast [48], the Ca^2+^-dependent formation of the ternary complex of ALG-2, AnxA11 and Sec31A may contribute to a unique and fine tuning of the ER to Golgi transport system in higher vertebrates. Helm *et al.* [22] have recently shown that specific depletion of luminal Ca^2+^ by cyclopiazonic acid (CPA), a reversible sarcoplasmic/endoplasmic reticulum Ca^2+^-ATPase (SERCA) inhibitor, inhibits ER to Golgi transport of vesicular stomatitis virus glycoprotein (VSV-G)-fused GFP and causes COPII proteins to accumulate in enlarged, intensified peripheral puncta in normal rat kidney cells. Surprisingly, overexpression of ALG-2 alone or the Sec31A Pro-rich region (PRR) alone caused no effects on the transport, but co-over-expression of both proteins exhibited prominent inhibitory effects [22]. It is interesting to see whether Pocket 3-occupied ALG-2 targets unknown regulator(s) in the ER to Golgi transport pathway or disrupt the linkage of Sec31A with AnxA11, which has been found to be a new modulator of the transport [23]. In conclusion, ALG-2 recognizes two types of motifs at different hydrophobic surfaces. The multivalent binding capacity of the dimeric ALG-2 protein should facilitate the modulation of various regulatory proteins by bridging them locally in a Ca^2+^-dependent fashion.

## 4. Experimental Section

### 4.1. Expression Plasmids

Expression plasmids of GST-fused peptides of ALG-2-binding sites of Sec31A and other proteins were constructed by inserting synthetic oligonucleotide blocks (NPPPPGFI, for instance, 5'-cggcaaatccaccgccacctggattcatcgcctcgg-3' and 5'-aggcgatgaatccaggtggcggtggatttgccgaac-3', Sec31A peptide coding region underlined) into the BglI site of pET42b(+) GST-SGG-linker-8xHis [27]. Construction of bacterial expression plasmids of GST-fused ALG-2 of wild-type (WT) and mutants (*E47A*/*E114A*, *ΔGF122*, *Y180A*) was described previously [27,28,39]. The mutant expressing GST-ALG-2^E47A^ and GST-ALG-2^E114A^ was obtained by mutagenesis using pGST-ALG-2 as a template. An expression plasmid of GFP-Sec31A was constructed by subcloning a 3.7-kb EcoRI/SmaI fragment of pFLAG-Sec31A [29] into the EcoRI/SmaI site of pmEGFP-C3, a derivative of pEGFP-C3 (Takara Bio/Clontech, Otsu, Japan) that expresses a monomeric form of EGFP [49]. Mutants of GST-ALG-2 and GFP-Sec31A were obtained by introducing mutations into their respective expression vectors with a QuikChange Site-Directed Mutagenesis kit (Agilent Technologies, Santa Clara, CA, USA) using a pair of oligonucleotide primers. See Table S2 for the sequences of oligonucleotides used in this study.

### 4.2. Purification of Recombinant Proteins

GST-8xHis fused peptides were first purified from *Escherichia coli* BL21(DE3) with TALON metal affinity beads (Takara Bio/Clontech) and then further purified with glutathione Sepharose beads (GE Healthcare Japan, Tokyo, Japan). *N*-terminal truncated ALG-2 (des3-20ALG-2) was expressed and affinity-purified essentially as described previously, except for using a column immobilizing a 15-residue ALG-2-binding oligopeptide (KQAPAPGWALFPSPG, a PLSCR3 peptide of F49W amino acid substitution) [28]. After dialysis against buffer TEG (20 mM Tris–HCl, pH 7.5, 10 μM EDTA, 10 μM EGTA), des3-20ALG-2 was concentrated with an ultrafiltration membrane (Amicon Ultra, Merck Millipore, Billerica, MA, USA) and applied to a Superdex 75 gel filtration column (GE Healthcare Japan, Tokyo, Japan) equilibrated with 10 mM Tris–HCl, pH 7.5, 150 mM NaCl and 1 mM EDTA. Peak fractions were collected, dialyzed against buffer TEG and concentrated as described above.

### 4.3. Crystallization

Crystallization conditions were first screened with an automated large-scale protein-crystallization system named PXS [50] at the Structural Biology Research Center in the High Energy Accelerator Research Organization (KEK, Tsukuba, Japan) and then further optimized manually. Concentrated des3-20 ALG-2 protein (11 mg/mL protein) was mixed with the 12-residue synthetic Sec31A oligopeptide (NPPPPGFIMHGN, >90% purity, GL Biochemicals, Shanghai, China) at a molar ratio of 1:2 and crystallized by the hanging drop method using 100 mM sodium cacodylate, pH 6.0, 20% 2-methyl-2,4-pentanediol (MPD) and 50 mM zinc acetate at 20 °C.

### 4.4. Data Collection, Structure Determination and Refinement

Data were collected using the beamline of PF-AR NE3A (KEK, Tsukuba, Japan) at a wavelength of 1.2821 Å with an ADSC Quantum 270 CCD detector under cryogenic conditions with crystals soaked in a cryoprotectant solution containing 20% glycerol and cooled to 100 K in a nitrogen gas stream. Diffraction data were integrated and scaled with the HKL2000 package [51]. The crystal structure was solved by the molecular replacement method using the program MOLREP [52] with the structure of the Ca^2+^-free form of des3-20ALG-2 (PDB code: 2ZND) as a search model. The model was refined first with REFMAC5 [53] and then with phenix.refine [54] in cycles of restrained refinement of the molecular model alternating with manual building using Coot [55]. Graphical representations were generated using PyMOL (v.1.3; Schrödinger, New York, NY, USA).

### 4.5. Cell Culture and DNA Transfection

HEK293T and ALG-2-knockdown HEK293 (HEK293/ALG-2_KD_) cells that were established by constitutive expression of the short hairpin RNA specific for ALG-2 [36] were cultured in DMEM (Nissui, Tokyo, Japan) supplemented with 4 mM glutamine, 10% fetal bovine serum (FBS), 100 units/mL penicillin and 100 μg/mL streptomycin at 37 °C under humidified air containing 5% CO_2_. For DNA transfection, cells were seeded and cultured for one day, and then, they were transfected with the expression plasmid DNAs by the conventional calcium phosphate precipitation method. Cultured cells were harvested and washed in PBS (137 mM NaCl, 2.7 mM KCl, 8 mM Na_2_HPO_4_ and 1.5 mM KH_2_PO_4_, pH 7.4) for biochemical analysis.

### 4.6. GST-ALG-2 Pulldown

GST-ALG-2 pulldown assays were performed as described previously [56] using the cleared lysate of HEK293/ALG-2_KD_ cells. Briefly, cells were lysed with lysis buffer L (20 mM HEPES–NaOH, pH 7.4, 142.5 mM KCl, 1.5 mM MgCl_2_, 0.2% Nonidet P-40) containing protease inhibitors (0.1 mM Pefabloc, 3 µg/mL leupeptin, 1 µM E-64, 1 µM pepstatin and 0.1 mM phenylmethylsulfonyl fluoride). The supernatant obtained by centrifugation at 12,000× *g* (cleared cell lysate) was incubated with glutathione Sepharose beads immobilizing GST-fusion proteins in the presence of 100 μM CaCl_2_ or 2 mM EGTA at 4 °C overnight, and the beads were washed three times with lysis buffer containing 100 μM CaCl_2_ or 2 mM EGTA. Proteins bound to the beads were subjected to SDS-PAGE followed by Western blotting using antibodies against Sec31A [23] and ALIX (CVL-PAB0204, Covalab, Lyon, France). Signals were detected by the chemiluminescence method using Super Signal West Pico Chemiluminescent Substrate (Thermo Fisher Scientific, Rockford, IL, USA) and analyzed with LAS-3000mini (Fuji Film, Tokyo, Japan).

### 4.7. Far-Western

After purified GST-fused ALG-2-binding peptides had been resolved by SDS-PAGE, proteins transferred to polyvinylidene fluoride (PVDF) membranes (Immobilon-P, Merck Millipore, Billerica, MA, USA) were stained with a reversible protein stain kit (Thermo Fisher Scientific, Waltham, MA, USA), and blot images were captured with a scanner (EPSON GT-X970, Seiko Epson, Suwa, Japan). Then, membranes were destained according to the manufacturer’s instructions and probed with biotin-labeled ALG-2 (bio-ALG-2), essentially as described previously [27,56]. Chemiluminescence signals were analyzed with LAS-3000mini, followed by quantification with an image analysis software, Image J.

### 4.8. Co-Immunoprecipitation Assays

One day after HEK293T cells had been transfected with expression plasmids for GFP-fused Sec31A proteins, harvested cells were lysed with lysis buffer L containing protease inhibitors. Cleared cell lysates were incubated with antiserum against GFP (A-6455, Invitrogen/Molecular Probes, Carlsbad, CA, USA) in the presence of 10 μM CaCl_2_, followed by incubation with Dynabeads Protein G (Invitrogen/Life Technologies Japan, Tokyo, Japan), as described previously [56]. Proteins bound to the beads were subjected to WB using a mouse monoclonal antibody against GFP (clone B-2, Santa Cruz Biotechnology, Santa Cruz, CA, USA) and rabbit polyclonal antibody against ALG-2 [12] and then subjected to FW with bio-ALG-2.

### 4.9. Miscellaneous

The LIGPLOT program was used for automatic generation of 2D ligand-protein interaction diagrams [57]. Homology-based 3D-structure modeling of human Sec31A and AnxA11 was performed by SWISS-MODEL [58,59]. Alignment of Sec31 sequences was performed first with CLUSTAL Omega [60,61] and then manually adjusted. Color-coded surface representation of ALG-2 chain B (Figure 2) was based on amino acid scale: normalized consensus hydrophobicity scale [62].

## Supplementary Materials

Supplementary materials can be found at http://www.mdpi.com/1422-0067/16/02/3677/s1.

## Abbreviation

aa: amino acids
Anx: annexin
bio-ALG-2: biotin-labeled ALG-2
COPII: coat protein complex II
ESCRT: endosomal sorting complex required for transport
FW: far-Western
GFP: green fluorescent protein
GST: glutathione-*S*-transferase
IP: immunoprecipitation
PEF: penta-EF-hand
PPII: polyproline helix type II
WB: Western blotting
